# Effect of Contemporary Bariatric Surgical Procedures on Type 2 Diabetes Remission. A Population-Based Matched Cohort Study

**DOI:** 10.1007/s11695-016-2103-6

**Published:** 2016-02-27

**Authors:** Martin C. Gulliford, Helen Pascale Booth, Marcus Reddy, Judith Charlton, Alison Fildes, A. Toby Prevost, Omar Khan, for the King’s Bariatric Surgery Study Group

**Affiliations:** 1 King’s College London, London, UK; 2 NIHR Biomedical Research Centre at Guy’s and St Thomas’ NHS Foundation Trust, London, UK; 3 St George’s Hospital, London, UK

**Keywords:** Bariatric surgery, Type 2 diabetes mellitus, Primary care, Electronic health records, Antidiabetes drugs

## Abstract

**Objective:**

The objective of the study is to evaluate the effect of gastric banding, gastric bypass and sleeve gastrectomy on medium to long-term diabetes control in obese participants with type 2 diabetes mellitus.

**Research Design and Methods:**

Matched cohort study using primary care electronic health records from the UK Clinical Practice Research Datalink. Obese participants with type 2 diabetes who received bariatric surgery from 2002 to 2014 were compared with matched control participants who did not receive BS. Remission was defined for each year of follow-up as HbA1c <6.5 % and no antidiabetic drugs prescribed.

**Results:**

There were 826 obese participants with T2DM who received bariatric surgery including adjustable gastric banding (LAGB) 220; gastric bypass (GBP) 449; or sleeve gastrectomy (SG) 153; with four procedures undefined. Mean HbA1c declined from 8.0 % before BS to 6.5 % in the second postoperative year; proportion with HbA1c <6.5 % (<48 mmol/mol) increased from 17 to 47 %. The proportion of patients in remission was 30 % in the second year, being 20 % for LAGB, 34 % for GBP and 38 % for SG. The adjusted relative rate of remission over the first six postoperative years was 5.97 (4.86 to 7.33, *P* < 0.001) overall; for LAGB 3.32 (2.27 to 4.86); GBP 7.16 (5.64 to 9.08); and SG 6.82 (5.05 to 9.19). Rates of remission were maintained into the sixth year of follow-up.

**Conclusions:**

Remission of diabetes may continue for up to 6 years after bariatric surgical procedures. Diabetes outcomes are generally more favourable after gastric bypass or sleeve gastrectomy than LAGB.

## Introduction

The potential role of bariatric surgery in the prevention [1, 2] and treatment [3] of diabetes in individuals with severe obesity is increasingly recognised [4]. A systematic review found that use of bariatric surgery in obese patients with type 2 diabetes was associated with remission of diabetes in approximately 70 % of patients over the first 2 years following surgery [5]. Most randomised trials have evaluated outcomes for small samples of patients, with outcomes reported more than 2 years following surgery only for a few patients [6, 7]. There are similarly few non-randomised studies that have reported on outcomes of diabetic patients more than 2 years after bariatric surgery [8]. The largest and longest-running study, the Swedish obese subjects (SOS) study, reported rates of remission of 38 % at 10 years and 30 % at 15 years follow-up, with fewer microvascular and macrovascular complications of diabetes in patients receiving surgery [9]. Although this study does provide important information, it should be noted that the majority of participants in the SOS cohort received the vertical band gastroplasty procedure—which is no longer widely utilised. There is a dearth of information on the long-term outcome data for currently used bariatric surgical procedures, including gastric banding and gastric bypass procedures, with data being especially limited for sleeve gastrectomy. In addition, most studies have been carried out in research centres and there are few pragmatic studies of the outcomes of patients treated in usual clinical practice settings.

We have utilised primary care electronic health records from a large database of UK family practices in order to perform a population-based comparison study of patients receiving currently used bariatric surgical procedures with matched obese subjects who did not undergo surgery. In a previous report, we evaluated the effect of bariatric surgery in the prevention of diabetes in obese subjects [1, 10]. In the present study, we aimed to compare the effect of the three different bariatric surgical procedures, gastric banding, gastric bypass and sleeve gastrectomy, on diabetes remission, and to evaluate the extent to which rates of remission were maintained over a maximum of 6 years of follow-up.

## Methods

### Data Source

Participants were sampled from the UK Clinical Practice Research Datalink (CPRD). The CPRD is a continuously-updated collection of primary care electronic health records from 1990 to the present. The CPRD presently draws data from approximately 680 general medical practices across the UK with a registered population of more than 5 million that is socio-demographically representative of the UK general population [11]. Data collected into CPRD include all consultations, referrals and hospital letters, drug prescriptions and medical tests for registered patients. Research quality data in CPRD conform to defined standards for research quality and the validity of CPRD clinical diagnoses has been documented [12]. Data were extracted from the May 2013 release of CPRD with potential follow-up to 30th April 2014.

### Participants

For the present study, participants aged 20 years or over were selected if they had a diagnosis of bariatric surgery recorded more than 12 months after the start of their CPRD record, had a recorded body mass index (BMI) record ≥30 Kg/m^2^ and were diagnosed with type 2 diabetes mellitus before the date of surgery. A minimum BMI value of 30 Kg/m2 was employed to ensure that all participants were obese but some BMI records dated from several years before operation and might not reflect pre-operative BMI. Baseline BMI values were recorded a median of 1.6 years (interquartile range 0.6 to 5.4 years) before surgery. Bariatric surgery procedures were identified using medical codes for laparoscopic adjustable gastric banding (LAGB), gastric bypass (GBP) or sleeve gastrectomy (SG). A diagnosis of type 2 diabetes was taken as the earlier of a medical diagnosis of diabetes, a prescription for antidiabetes medicines, or an HbA1c value >6.5 % (48 mmol/mol). Participants who were diagnosed with polycystic ovary syndrome and prescribed diabetes medicines, but not diagnosed with diabetes were excluded. Participants who were ever diagnosed with gestational diabetes were also excluded from these analyses.

Control participants were selected who never had bariatric surgery recorded, but were obese and had type 2 diabetes mellitus diagnosed before the index date. As the distribution of BMI differed greatly between BS cases and the CPRD obese population, control participants were individually matched with cases using nearest neighbour matching on BMI, age, sex and index year. The index date for controls was the date of the first BMI record on which they entered their highest recorded BMI category. Participant records ended if they terminated their registration with a CPRD general practice; if their general practice ended participation in CPRD; if the latest data collection date was reached; or if the patient died.

There were 4793 obese participants with bariatric surgery recorded, 1324 were excluded because the index code was within 12 months of the start of the record, 14 were excluded with age less than 20 years, 401 were excluded because their BMI was less than 30 or no values were recorded before surgery, 2176 were excluded as non-diabetic, leaving 878 obese participants with type 2 diabetes of whom 52 were excluded with gestational diabetes ever recorded. There were then 826 obese participants with type 2 diabetes diagnosed before surgery who were matched with 826 obese diabetic controls who did not receive surgery.

Participants were registered at 360 general practices, of which 92 % continued their participation in CPRD until 2013 or later; there were 69 participants that ended their registration with a CPRD practice before 2013. There were 20 participants who died during the period of follow-up.

### Main Measures and Analysis

HbA1c records and prescriptions for oral hypoglycaemic drugs and insulin were evaluated for BS cases and controls. The person time for each participant was divided into study years from 3 years before the procedure to 6 years after the procedure. This allowed us to conduct an interrupted time-series analysis. Follow-up was censored at 6 years because few cases remained under follow-up for sleeve gastrectomy and gastric bypass. The highest HbA1c value and the total number of diabetes prescriptions were evaluated in each study year. For each year of follow-up, participants were classified as being in remission if the maximum HbA1c value recorded in year was <6.5 % and there were no diabetes prescriptions issued in the year. Relative rates were estimated for each year following the BS procedure by using a Poisson model with person time as the exposure. A model was fitted to evaluate the effect of group (BS or control) and time after surgery, included as indicator variables for each postoperative year (13). Confounders included age, gender, BMI, quartile of diabetes duration before surgery, whether coronary heart disease (CHD), stroke or depression were diagnosed before the index date, whether blood pressure (BP) was ≥140/90 mmHg or serum total cholesterol ≥5 mmol/L, and whether antihypertensive drugs and lipid-lowering drugs were prescribed before the index date, and current smoking recorded before the index date.

## Results

Baseline characteristics of the bariatric surgery participants and controls are shown in Table 1. Bariatric surgery participants and controls were generally similar with respect to age, gender and index year but the baseline BMI was higher in the BS participants. BS participants also had longer duration of diabetes with a median duration of 5.5 years since diagnosis, compared with 3.1 years for controls. BS participants were more likely to be treated with statins and antihypertensive drugs, with lower blood pressure and cholesterol values, and were less likely to be current smokers.

**Table 1:** Baseline characteristics of bariatric surgery participants and controls. Figures are frequencies (column percent) except where indicated

	BS participants	Control participants	*P* value
Number	826	826	
Female	542 (66)	524 (63)	0.390
Age (years, mean SD)	50.0 (9.6)	49.1 (13.8)	0.118
Body mass index (Kg/m^2^)	46.7 (8.3)	44.2 (6.5)	<0.001
Index year (median, interquartile range)	2011 (2009 to 2012)	2011 (2010 to 2012)	0.495
Diabetes duration (years, median, interquartile range)	5.5 (2.4 to 9.3)	3.1 (0.3 to 7.1)	<0.001
Antihypertensive therapy	632 (77)	517 (63)	<0.001
Blood pressure >140/90 mmHg	247 (30)	287 (35)	0.027
Statin therapy	579 (70)	451 (55)	<0.001
Total cholesterol >5 mmol/L	217 (26)	257 (31)	<0.001
Current smoking	117 (14)	189 (23)	<0.001

There were 220 (27 %) BS patients who received LAGB, 449 (54 %) received gastric bypass (GBP) procedures, 153 (19 %) received sleeve gastrectomy (SG). The type of procedure was undefined for four participants with more than one operation type coded on the index date. LAGB procedures were used in clinical practice at an earlier time having a median index year of 2009, compared with 2011 for gastric bypass or sleeve gastrectomy.

Tables 2 and 3 show data for the number of participants contributing person time to the analysis, from 3 years before to 6 years after the BS procedure. The proportion of participants contributing person time in each postoperative year declined rapidly because more than 50 % of procedures were within the last 4 years and only 13 % of participants contributed person time in the sixth year of follow-up. Participants receiving LAGB contributed a higher proportion of person time at long follow-up. The proportion of participants with HbA1c values recorded generally ranged between 60 and 80 % (Tables 2 and 3). At the time of surgery, slightly more BS patients had HbA1c values recorded, but at longer durations of follow-up, HbA1c recording was more complete in controls than BS patients.

**Table 2:** Diabetes remission before and after surgery for bariatric surgery (BS) participants and controls. Remission was defined, by study year, as HbA1c <6.5 % (<48 mmol/mol) and no diabetes prescriptions in year. Figures are frequencies (column percent) except where indicated

	Years from procedure								
	−3 to −2	−2 to −1	−1 to 0	0 to 1	1 to 2	2 to 3	3 to 4	4 to 5	5 to 6
Controls									
Participants contributing PT	399	567	826	826	733	464	272	161	101
HbA1c recorded	278 (70)	388 (68)	626 (76)	612 (74)	536 (73)	309 (67)	185 (68)	113 (70)	81 (80)
In diabetes remission	20 (5)	29 (5)	32 (4)	34 (4)	27 (4)	27 (4)	9 (3)	8 (5)	6 (6)
All BS cases									
Participants contributing PT	692	752	826	826	674	499	336	212	109
HbA1c recorded	506 (80)	607 (81)	698 (85)	663 (80)	499 (74)	321 (64)	321 (64)	125 (59)	69 (63)
In diabetes remission	26 (4)	32 (4)	39 (5)	177 (21)	204 (30)	124 (25)	71 (21)	44 (21)	18 (17)
RR (95 % CI)^a^	Ref.	Ref.	Ref.	4.66 (3.80 to 5.73)	7.16 (5.79 to 8.86)	6.68 (5.21 to 8.56)	6.17 (4.60 to 8.28)	7.05 (5.03 to 9.88)	5.90 (3.72 to 9.34)

*RR* relative rate, *BS* bariatric surgery, *PT* person time

^a^Adjusted for age, gender, BMI, diabetes duration quartile, prevalent CHD, stroke, depression, smoking status, elevated total cholesterol, high blood pressure, use of antihypertensive drugs and statins, and year of procedure

**Table 3:** Diabetes remission before and after surgery for BS participants by procedure type. Remission was defined, by study year, as HbA1c <6.5 % (<48 mmol/mol) and no diabetes prescriptions in year. Figures are frequencies (column percent) except where indicated

	Years from procedure								
	−3 to −2	−2 to −1	−1 to 0	0 to 1	1 to 2	2 to 3	3 to 4	4 to 5	5 to 6
LAGB									
Participants contributing PT	175	197	220	220	201	177	143	115	81
In diabetes remission	7 (4)	9 (5)	7 (3)	15 (7)	40 (20)	32 (18)	28 (20)	21 (18)	11 (14)
RR (95 % CI)^a^	Ref.	Ref.	Ref.	1.40 (0.80 to 2.44)	4.16 (2.84 to 6.11)	4.01 (2.56 to 6.29)	4.34 (2.73 to 6.89)	4.06 (2.44 to 6.75)	3.19 (1.70 to 5.97)
Gastric bypass									
Participants contributing PT	344	413	449	449	361	249	155	81	17
In diabetes remission	13 (4)	14 (3)	19 (4)	112 (25)	122 (34)	70 (28)	33 (21)	18 (22)	3 (18)
RR (95 % CI)^a^	Ref.	Ref.	Ref.	5.83 (4.55 to 7.48)	8.55 (6.67 to 11.0)	7.77 (5.82 to 10.4)	6.27 (4.36 to 9.02)	8.78 (5.68 to 13.6)	11.7 (5.15 to 26.6)
Sleeve gastrectomy									
Participants contributing PT	107	138	153	153	109	70	36	14	10
In diabetes remission	6 (6)	8 (6)	13 (9)	48 (31)	41 (38)	22 (31)	9 (25)	5 (36)	4 (40)
RR (95 % CI)^a^	Ref.	Ref.	Ref.	6.21 (4.59 to 8.41)	6.21 (4.59 to 8.41)	6.96 (4.65 to 10.4)	6.15 (3.27 to 11.6)	8.63 (4.07 to 18.3)	8.63 (4.07 to 18.3)

*RR* relative rate, *BS* bariatric surgery, *LAGB* laparoscopic adjustable gastric banding, *PT* person time

^a^Adjusted for age, gender, BMI, diabetes duration quartile, prevalent CHD, stroke, depression, smoking status, elevated total cholesterol, high blood pressure, use of antihypertensive drugs and statins, and year of procedure

**Fig. 1:**
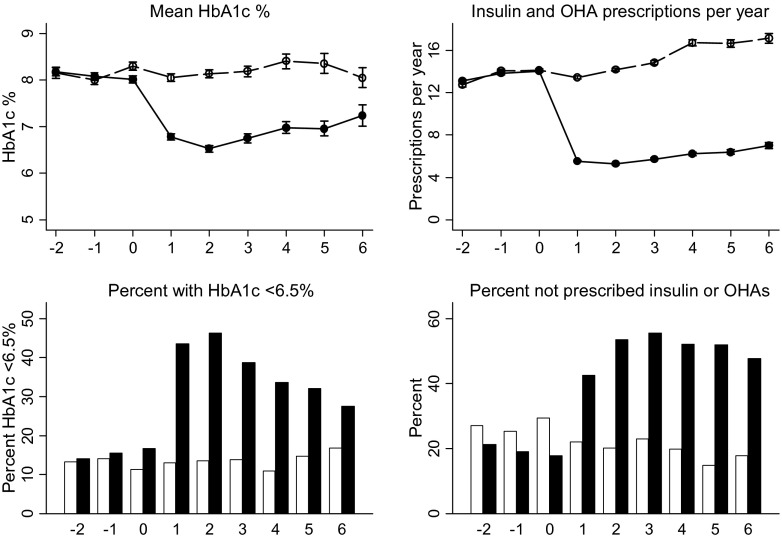
Changes by year from bariatric surgery in mean (SE) HbA1c and mean (SE) diabetes prescriptions per year. *Black symbols*, bariatric surgery participants; *hollow symbols*, control participants. *OHA* oral hypoglycaemic agents

Trends in mean HbA1c and proportion with HbA1c <6.5 % (<48 mmol/mol) are shown by year following surgery in Fig. 1. The mean HbA1c value in the year before the index date was 8.0 % (64 mmol/mol) in BS cases and 8.3 % (67 mmol/mol) in controls. The mean HbA1c value in BS cases declined to 6.8 % (51 mmol/mol), 6.5 % (48 mmol/mol) and 6.8 % (51 mmol/mol) in the first 3 years following surgery, but remained unchanged at 8.1 % (65 mmol/mol), 8.2 % (66 mmol/mol) and 8.2 % (66 mmol/mol), respectively in controls. The proportion of BS cases with HbA1c values <6.5 % (<48 mmol/mol) was 17 % before operation, increasing to 44, 47 and 39 % in the first three postoperative years. No consistent trend in the proportion of controls with HbA1c <6.5 % (<48 mmol/mol) was observed. Trends in the proportion of participants without antidiabetes drug prescriptions and the mean number of drug prescriptions per participant year are also shown for BS cases and controls in Fig. 1. The proportion of BS cases without antidiabetic drugs or insulin prescriptions increased from 15 % before operation to 41, 53 and 55 % in the first 3 years after surgery, while the opposite trend was observed in controls.

The proportion of BS cases in remission was 5 % before operation, increasing to 21, 30 and 25 % in the first 3 years after surgery (Table 2), while the proportion of controls in remission tended to remain constant over time. In the first year after surgery, the proportion of participants in remission was lower for LAGB patients (7 %, 95 % confidence interval 4 to 11 %) than for patients receiving either gastric bypass (25 %, 21 to 29 %) or sleeve gastrectomy (31 %, 24 to 39 %) (Table 3). When remission was evaluated only including participants with HbA1c values recorded in a given year, then the proportion of participants in remission for the first and subsequent years following the procedure was 27, 41, 39, 33, 35 and 26 %; the equivalent figures for controls were 6, 5, 7, 5, 7 and 7 %. The proportion of participants with either HbA1c <6.5 %, or not taking medications, was higher than the proportion of participants in remission, which required both criteria to be met. Of the 744 person years in remission among bariatric surgery cases, 175 (24 %) were not in complete remission (HbA1c <6.0 %). The proportion of BS participants in complete remission in the second year after the procedure was 26 %.

Multivariable adjusted analyses were conducted using participant years as observations (Table 2 and 3). The adjusted relative rate of diabetes remission across all types of bariatric surgery, relative to person time without surgery, was 4.66 (95 % confidence interval 3.80 to 5.73) in the first year after surgery increasing to 7.16 (5.79 to 8.86) in the second year. By the sixth year of follow-up, the adjusted relative rate was 5.90 (3.72 to 9.34). In participants receiving LAGB, compared with all controls, the adjusted relative rate of diabetes remission was not elevated in the first postoperative year but increased to 4.16 (2.84 to 6.11) in the second postoperative year. The rate of remission remained elevated for the sixth year of follow-up (3.19, 1.70 to 5.97). For either gastric bypass or sleeve gastrectomy, the rate of diabetes remission was increased in the first year following the procedure (GBP 5.83, 4.55 to 7.48; SG 6.21, 4.59 to 8.41) and remained elevated until the end of the sixth year of follow-up (GBP 11.7, 5.15 to 26.6; SG 8.53, 3.75 to 19.4). For these procedures, adjusted rate ratios were higher than for LAGB, though confidence intervals overlapped. There were small numbers of observations for the later years of follow-up for gastric bypass and sleeve gastrectomy. Associations of confounders with remission were generally of small magnitude except for a graded association of duration of diabetes, with the highest quartile of diabetes duration (diabetes diagnosed more than 8.6 years before surgery) being associated with reduced relative risk of remission of 0.23 (95 % confidence interval 0.16 to 0.33, *P* < 0.001).

Table 4 presents adjusted rate ratios for diabetes remission for combining the 6 years of follow-up. The adjusted relative rate of diabetes remission after bariatric surgery compared with controls was 5.97 (95 % confidence interval 4.86 to 7.33, *P* < 0.001). The three types of bariatric surgery each resulted in a significantly higher rates of diabetes remission compared with controls (all *P* < 0.001). However, the rate of remission was lower after LAGB 3.32 (2.27 to 4.86), compared with gastric bypass (7.16, 5.64 to 9.08) or sleeve gastrectomy (6.82, 5.05 to 9.19). The relative rate of diabetes remission was slightly higher in men compared to women. The relative rate of diabetes remission increased with increasing BMI category, being 6.74 (5.29 to 8.58) with BMI ≥40 Kg/m^2^ but 4.23 (95 % CI 1.98 to 9.03) at BMI ≥30 Kg/m^2^. The relative rate of diabetes remission was highest in participants aged 35 to 54 years.

**Table 4:** Association of bariatric surgery with diabetes remission by age group, gender, BMI category and procedure type

		Adjusted^a^ rate ratio (95 % confidence interval)	*P* value
All BS procedures		5.97 (4.86 to 7.33)	<0.001
Type of procedure^b^	Laparoscopic gastric banding	3.32 (2.27 to 4.86)	<0.001
	Gastric bypass	7.16 (5.64 to 9.08)	<0.001
	Sleeve gastrectomy	6.82 (5.05 to 9.19)	<0.001
Sex	Men	7.70 (5.26 to 11.3)	<0.001
	Women	5.32 (4.16 to 6.81)	<0.001
Baseline BMI category (Kg/m^2^)	30–34.9	4.23 (1.98 to 9.03)	<0.001
	35–39.9	3.59 (2.16 to 5.97)	<0.001
	≥40	6.74 (5.29 to 8.58)	<0.001
Age group (years)	20 to 34	5.38 (2.81 to 10.3)	<0.001
	35 to 54	6.69 (5.05 to 8.86)	<0.001
	≥55	5.52 (3.91 to 7.80)	<0.001

*BMI* body mass index, *BP* blood pressure, *BS* bariatric surgery, *CHD* coronary heart disease

^a^Adjusted for age, gender, BMI, diabetes duration quartile, prevalent CHD, stroke, depression, smoking status, elevated total cholesterol, high blood pressure, use of antihypertensive drugs and statins, and year of procedure

^b^Three cases with more than one procedure coded on index date were excluded

## Discussion

Our study demonstrates that bariatric surgery was associated with a sixfold increase in diabetes remission compared with no bariatric surgery and that patients receiving either gastric bypass or sleeve gastrectomy procedures, rather than gastric banding, showed earlier onset and higher rates of diabetes remission. Changes in mean HbA1c in our study were similar to those reported in previous studies [13]; rates of diabetes remission after surgery in the present study were significantly lower than those reported in published reviews [6, 14, 15] and in UK National Bariatric Surgery Registry (NBSR) data, which reported 80 % of patients to be in remission 3 years post-surgery [16]. A retrospective cohort study by Arterburn et al. [17] found partial diabetes remission rates of 47, 73 and 77 % at 1, 3 and 5 years after gastric bypass surgery. The reasons for the differences in observed remission rates are not clear, although patients in our study had a slightly longer history of diabetes, which Arterburn et al. found to reduce likelihood of remission. The proportion of patients in remission declined over time following surgery and there was biochemical evidence that individual patients showed relapse of diabetes after a period of remission. It should be noted that we used a stringent definition of remission that required a normal HbA1c value to be recorded in each year and patients who had missing values for HbA1c were assumed not to be in remission. A recent paper using CPRD data found overall 28 % diabetes remission, based on ‘remission’ recorded by family physicians [18]. When remission rates were estimated including only participants with HbA1c values recorded in year, the proportion of participants in remission was higher. Missing HbA1c values may have been related to diabetes control, possibly with less frequent recording in patients who are in remission or have good control. In clinical practice, some patients may be continued on metformin therapy even in the setting of apparent biochemical remission and underestimation of remission may also occur if patients receive repeat prescriptions for medicines they no longer require. Conversely, relapse after previous remission may not be detected if blood glucose control is not evaluated. Even allowing for these limitations, this pragmatic study raises questions concerning whether the results achieved in routine practice may be as favourable as those reported from research studies.

Previous studies suggest that gastric bypass is more effective for the treatment of diabetes than LAGB [5, 14]. This finding is replicated in our study, and in addition, we found that sleeve gastrectomy was superior to gastric banding and similarly as effective as gastric bypass in leading to diabetes remission. This result contradicts a randomised controlled trial in 60 patients by Lee et al., who demonstrated superior glycaemic control in diabetic patients undergoing mini-gastric bypass as opposed to sleeve gastrectomy [19]. Although the majority of sleeve gastrectomies occurred in the later part of the study and pragmatic observational studies are more susceptible to bias than results from randomised trials, our results do suggest that sleeve gastrectomy may be as effective as gastric bypass for diabetes remission. Previous studies have shown higher diabetes remission rates in patients with greater post-surgery weight loss [5]. Unfortunately, we were unable to assess this recording of weight over time in primary care is very limited, even in this high-interest sub-group of patients. Our results offer some suggestion that the effect of bariatric surgery may decline over time; however, we accept the low overall follow-up rate and the fact that the majority of long-term follow-up data is from LABG does limit the conclusions which can be drawn.

This study has several limitations. The available duration of follow-up was generally shorter for gastric bypass and sleeve gastrectomy procedures than for patients who received LAGB as a consequence of the changes in popularity of these procedures over time. In addition, control patients were not treated using a standardised weight loss programme, and it is possible that diabetes management in the two groups also differed. Surgery patients may have had better access to specialist diabetes care in the run-up to their surgery. While cases and controls were matched, there were residual differences for some variables including duration of diabetes, which was longer in the surgical group. However, our analyses were adjusted for potential confounders. Perhaps, most importantly, there was a persistent issue of missing and inconsistently recorded data as detailed previously. The analyses were conducted on an ‘intention to treat’ basis, and though small numbers of participants may have had further bariatric procedures, these were not excluded.

In conclusion, this is one of the largest prospective pragmatic studies on the impact of bariatric surgery on diabetes outcomes and demonstrates that bariatric surgery (and in particular gastric bypass and sleeve gastrectomy) may facilitate diabetes control in obese patients treated in routine primary care settings. The three most commonly used surgical techniques were associated with increased rates of remission, improved blood glucose control and reduced use of antidiabetes medications for type 2 diabetes mellitus over a maximum of 6-year follow-up period. Rates of remission tended to decline over time and there was biochemical evidence of relapse in some participants. There remains a clear need for longer-term follow-up studies to assess the clinical- and cost-effectiveness of bariatric surgery on diabetes outcomes, including assessments of adverse events and safety.

## Abbreviations

GBP: Gastric bypass
LAGB: Laparoscopic adjustable gastric banding
SG: Sleeve gastrectomy
T2DM: Type 2 diabetes mellitus
